# Massive Data Management and Sharing Module for Connectome Reconstruction

**DOI:** 10.3390/brainsci10050314

**Published:** 2020-05-22

**Authors:** Jingbin Yuan, Jing Zhang, Lijun Shen, Dandan Zhang, Wenhuan Yu, Hua Han

**Affiliations:** 1 School of Automation, Harbin University of Science and Technology, Harbin 150080, China; yuanjingbin@hotmail.com (J.Y.); zhangjing@hrbust.edu.cn (J.Z.); 2 Research Center for Brain-inspired Intelligence, Institute of Automation, Chinese Academy of Sciences, Beijing 100190, China; dandan.zhang@ia.ac.cn (D.Z.); wenhuan.yu@ia.ac.cn (W.Y.); 3 The National Laboratory of Pattern Recognition, Institute of Automation, Chinese Academy of Sciences, Beijing 100190, China; 4 Center for Excellence in Brain Science and Intelligence Technology, Chinese Academy of Sciences, Shanghai 200031, China; 5 The School of Future Technology, University of Chinese Academy of Sciences, Beijing 100049, China

**Keywords:** connectome, massive data management, distributed storage and retrieval, electron microscope image, segmentation result, image cache

## Abstract

Recently, with the rapid development of electron microscopy (EM) technology and the increasing demand of neuron circuit reconstruction, the scale of reconstruction data grows significantly. This brings many challenges, one of which is how to effectively manage large-scale data so that researchers can mine valuable information. For this purpose, we developed a data management module equipped with two parts, a storage and retrieval module on the server-side and an image cache module on the client-side. On the server-side, Hadoop and HBase are introduced to resolve massive data storage and retrieval. The pyramid model is adopted to store electron microscope images, which represent multiresolution data of the image. A block storage method is proposed to store volume segmentation results. We design a spatial location-based retrieval method for fast obtaining images and segments by layers rapidly, which achieves a constant time complexity. On the client-side, a three-level image cache module is designed to reduce latency when acquiring data. Through theoretical analysis and practical tests, our tool shows excellent real-time performance when handling large-scale data. Additionally, the server-side can be used as a backend of other similar software or a public database to manage shared datasets, showing strong scalability.

## 1. Introduction

The brain is the organ of thought, and as the most advanced part of the nervous system, it dominates all the activities of the body. To explore the brain, scientists have done much research and have a basic understanding of its functions. For example, MRI technology is adopted to study the connections between different gray matter areas of the brain on a macro scale (millimeter scale) [1]. Based on optical technology, the projection path of a single neuron axon and its connection with upstream and downstream neurons are studied at the mesoscopic scale (micron scale) [2,3]. To ensure that all parts of the brain work together and drive the body’s activities, the transmission of signals, and the connection between the large number of neurons is still a mystery. The significant number of neurons and the complex connections between them make the structure and function of the brain extremely complicated. One of the ways to explore the mechanism is to use the electron microscope (EM) to image sequence slices of the brain tissue and then draw the connectome of it [4,5,6]. It possesses one advantage that the imaging mode has enough high spatial resolution (nanoscale) to clearly distinguish the connections concerning the delicate structures of the neurons, such as connections between synapses.

**Figure 1:**
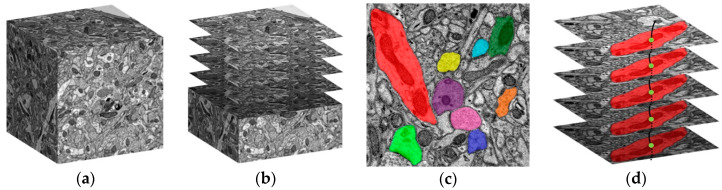
A schematic diagram of the sequential electron microscope images and the segmentation data that makes up a neuron. (**a**) A schematic diagram of the brain tissue block; (**b**) Sequential electron microscope (EM) images obtained from the brain tissue block; (**c**) Different colors represent different segments. Each segment represents a segmentation region of a neuron in the layer; (**d**) The segments on each layer form a neuron.

The reconstruction of the connectome usually requires a series of steps such as sample preparation, slicing, EM imaging, image registration, automatic segmentation, and proofreading. A schematic diagram of obtaining the sequence images by the electron microscope from a brain tissue block is shown in Figure 1a,b.

With the development of advanced technology and the expectation of reconstructing complete connectome, the volume of electron microscope images grows significantly. The difficulty of EM images management is increasing. For one, to ensure the continuity between sequence images and the limitation of the actual physical size of the neural structure, the thickness of the slice should be thinner and is generally 30–50 nm or even 5 nm. For another, in order to observe a more delicate structure, the spatial resolution of imaging is also required to be higher. With the development of EM imaging technology, it is possible to fulfill these requirements, such as the serial block-face scanning electron microscopy (SBF-SEM) [7], the focused-ion-beam scanning electron microscopy (FIB-SEM) [8,9], the automated tape-collecting ultramicrotome scanning electron microscopy (ATUM-SEM) [10,11], TEMCA [12], and the Zeiss MSEM [13,14]. As a consequence, the imaging speed is significantly improved and more image data can be obtained at an acceptable time. Thus, the volume of the image data obtained by the EM becomes more massive and can be up to terabytes (TB) and even petabytes (PB). Table 1 shows the reconstruction results over the years. The volume of reconstruction is increasing year by year, and the state of reconstruction gradually changes from sparse to dense. Therefore, large-scale image datasets will be more common in the future, with the excellent value for understanding the neuronal connection and the mechanism of signal transmission in our brain.

The large-scale EM images and the application of the automatic segmentation algorithm with high accuracy often bring a large number of reconstruction results (usually the segmentation data), such as Convolutional Neural Networks (CNN) [23,24]. The difficulty of managing segmentation results lies in its number and irregular shape. According to the physical size of neurons and fractal theory, the number of the segments (as shown in Figure 1c) in a volume of 1 mm^3^ brain tissue, maybe billions or even more. Irregular shape makes storage and retrieval difficult. Therefore, managing such big data during the reconstruction process to explore the mechanism of our brain is still a challenge to be faced. For this purpose, it is necessary to develop a big data management tool for researchers.

## 2. Related Work

In the field of connectomics, many excellent tools have been developed recently, such as CATMAID [25,26], which is inspired by online mapping applications. Knossos [15] and webKnossos [27] is for 3D image visualization and annotation. TrakEM2 [28] is an image-based program for morphological data mining and 3D modeling. EyeWire [29,30] is a game to crowdsource brain mapping. Mojo and Dojo [31] are the interactive proofreading tool for connectomics. Vast [32] is a volume annotation and segmentation tool. NeuTu [33] with a distributed versioned image-oriented data service (DVID) [34] is for collaborative, large-scale, segmentation-based connectome reconstruction.

Here, a management module is designed specifically for managing the large-scale data of neural structure in the field of connectomics, so that researchers can mine valuable information during the proofreading process. Firstly, the most critical and essential problem is the large-scale volume of data. In this paper, a distributed architecture with Hadoop [35] (Hadoop is an open-source distributed framework under the Apache foundation, which allows for the distributed processing of large datasets across clusters of computers using simple programming models. When the existing service cluster cannot meet the storage requirements, new server nodes can be added to the server cluster seamlessly and keep stable.) and HBase [36] (HBase is an open-source distributed database under the Apache foundation, which is the Hadoop database, a versioned, scalable, nonrelational database modeled for storing big data. It can achieve random, real-time read-write access.) is adopted on the server-side as the underlying support for large-scale data, it can be easily deployed in clusters and is scalable. Secondly, a standard interface is designed to encapsulate the underlying structure. In this case, the client can interact conveniently with the data in the distributed framework. Based on the standard interface, the server-side of the module can be used as the back end of other similar software based on client/server (C/S) architecture or a public database to manage shared datasets. Thirdly, for rapidly retrieving the required data, combined with the characteristics of HBase, different retrieval methods are designed for EM images and segmentation data. Finally, to further reduce the time delay of data acquisition in large-volume data, a three-level cache module is put forward on the client-side, combined with the characteristics of data. The paper only introduces the leading strategies and methods of the data management module. For specific implementation details and demos, please refer to “Appendix A”.

## 3. Materials and Methods

In order to support the storage of large-volume data and meet the requirements for browsing at the client-side, this section introduces the storage and retrieval strategies of the EM image and segmentation data at the server-side in detail. At the client-side, a three-level image cache module is designed to reduce delay time when acquiring data.

### 3.1. Image Data Model Design

In order to understand the brain more comprehensively, with the improvement of advanced technology, researchers hope to reconstruct larger brain tissue blocks or even the whole brain. As the number of brain tissue slices and the imaging resolution of the electron microscope increases, the volume of data grows massive as well. Terabyte (TB)- and even petabyte (PB)-level EM image data will become more common. However, the resolution of the users’ monitor is not so large (for example, 1920 × 1080 pixels). When browsing images, the size of the image to be displayed is usually determined by monitor resolution. Therefore, it is only necessary to get an image of the same size as the monitor resolution instead of the whole image. If the entire image is read into the computer’s memory for display, it will be time-consuming, and as the image size increases, the memory size becomes a limitation. 

**Figure 2:**
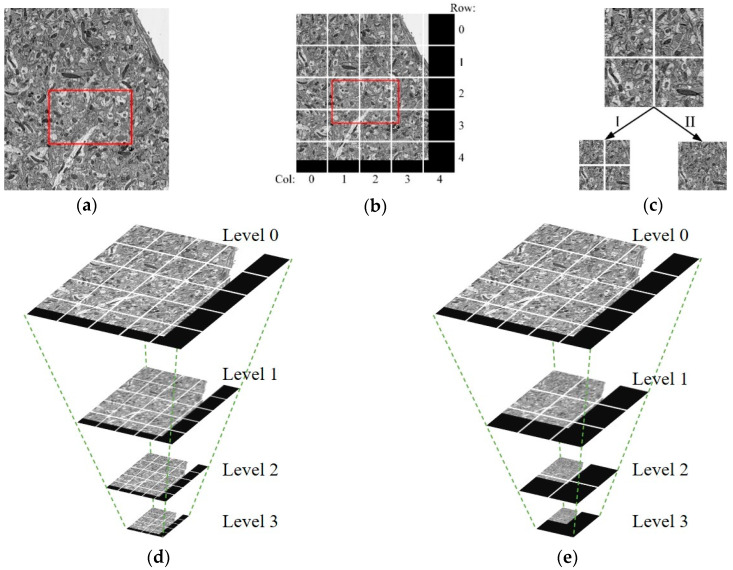
The designed image data model. (**a**) Suppose that the image in the viewport (the red box in the figure) is needed. Before the image is divided into blocks, the whole image needs to be obtained. (**b**) After the image is divided, only six small images need to be acquired; the block size is fixed, the undersized image blocks are filled with the black area. (**c**) Two downsampling methods: Method I: mipmap [37], before and after downsampling, the number of the image blocks is the same, but the image block size is reduced by four times; Method II: after downsampling, the image size remains unchanged, but the number of images is reduced. (**d)** The image pyramid generated by Method I used to obtain images at different resolutions. (**e**) The image pyramid generated by Method II; compared with Method I, the number of image blocks needed to be acquired in this method is less when acquiring low-resolution (high-level) images.

Therefore, in this paper, the image is divided into fixed-size blocks, as shown in Figure 2a,b. For the sake of the needs of observing neurons at different scales, such as observing the fine structure of neurons at high resolution and browsing the overall trend at low resolution, a multiresolution model is used to store EM images, called the image pyramid model, as shown in Figure 2d,e. The pyramid model, as shown in Figure 2d is generated by using the downsampling method, Method I, in Figure 2c. The pyramid model, as shown in Figure 2e, is generated by using the downsampling method, Method II in Figure 2c. As for the downsampling method in Figure 2c, after downsampling, the number of the image blocks is the same, but the image block size is reduced by four times than before. As for the downsampling method in Figure 2c, after downsampling, the image size remains unchanged, but the number of images is reduced. The number of image blocks needed to be acquired in Method II is less than that in Method I when acquiring low-resolution images, and it is significantly reduced. Therefore, the downsampling Method II is chosen to generate the image pyramid model. The maximum downsampling level is calculated as follows:

maxLevel = ⎡log_2_(max(rows, cols))⎤, (1)

where max(a, b) indicates taking the maximum value of a and b; rows and cols means total rows and columns of the image block.

### 3.2. Image Data Retrieval

**Figure 3:**
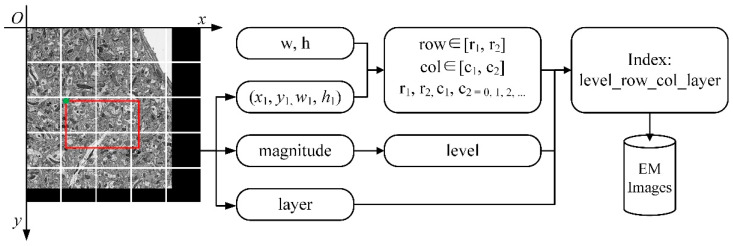
The flow of image retrieval. w and h represent the width and height of the image block, which has a fixed size, respectively. (x1, y1) is the upper left coordinate and size of the viewport (the red box in the figure); w1 and h1 are its sizes. w, h, and (x1, y1, w1, h1) are employed to calculate row and column numbers of image blocks (row, col). Magnitude is the scale of the image, and it is used to calculate the level of the image pyramid. The layer is the number of the sequence image. The level, row, col, and layer construct the indexes of image blocks for retrieval.

To obtain the image data to be displayed, the image blocks need to be indexed in the image pyramid. The key-value pair index is established for retrieval according to the spatial position of the image block and the level of the image pyramid. In addition to that, HBase is used as a database to store images, and the key of the index is the row key of the data in the database, named “layer_row_col_level”; the value of the index is the image block data at the corresponding position. For example, as shown in Figure 3, suppose “w” and “h” represent the width and height of the image block, (x1, y1, w1, h1) represents the viewport which is displayed at scale “magnitude” in layer “layer”. The transformation between magnitude and level is shown in Table 2. The start-row, end-row, start-column, and end-column of the image blocks that need to be read are calculated as follows:

start-row = ⌊y1/h⌋ = r1,(2)

end-row = ⌊(y1+ h1)/h⌋ = r2,(3)

start-column = ⌊x1/w⌋ = c1,(4)

end-column = ⌊(x1+ w1)/w⌋ = c2,(5)

row ∈ [r1, r2],(6)

col ∈ [c1, c2],(7)

Then through permutation and combination, the indexes of image blocks can be composed as “layer_row_col_level”. As the image volume increases, the retrieval speed will not be affected by the retrieval strategy. The method of key-value pair retrieval achieves constant time complexity O(1).

### 3.3. Image Cache

**Figure 4:**
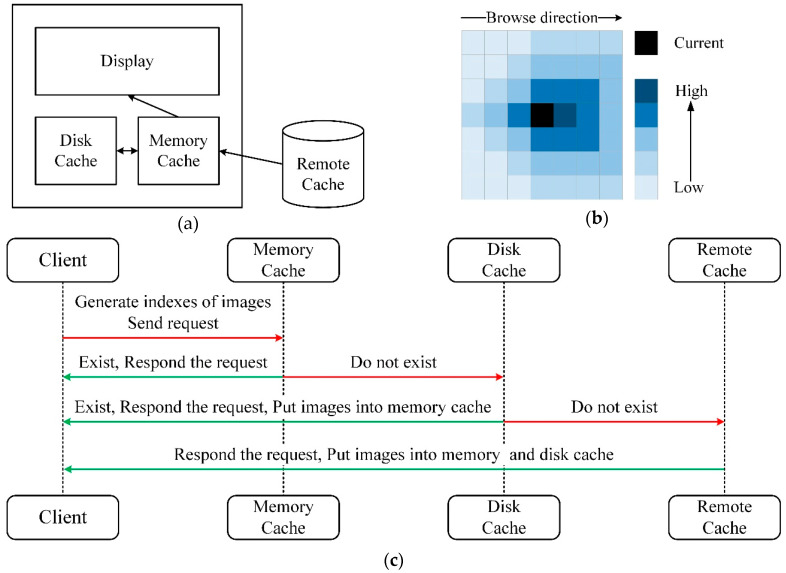
The image cache mechanism and operation process. (**a**) The architecture of the image cache module. The image cache falls into three levels. (**b**) The operation process of the image cache. The requested images must exist in the remote cache. (**c**) The schematic diagram of image cache priority. The black area represents the current view when browsing. In addition to the black area, the box with deeper color is of high priority, whereas on the contrary, the lighter color is of low priority.

In order to reduce the delay in acquiring images at the client-side, and give users a smoother image browsing experience, it is necessary to preload the image near the current browsing view of the client. Based on our image storage and retrieval methods as well as requirements for browsing EM images, a three-level cache module on the client is designed here, as shown in Figure 4a.

As shown in Figure 4c, when browsing, the client generates the indexes of images required by the current viewport. Firstly, the client sends a request to the memory cache. If the requested images exist in the memory cache, the images are directly returned to the client; if not, the request is sent to the disk cache. Similarly, if the data exists, the images are directly returned and put the images into the memory cache; otherwise, a request is sent to the remote cache. The client gets images from the remote cache and puts the images into the memory and the disk cache. During browsing, the image far away from the current browsing viewport in the cache will be deleted to avoid occupying too much memory or disk space.

It is beneficial to set up the cache priority of images, for firstly caching the images in the browsing direction near the current viewport. In Figure 4b, suppose the black area is the currently displayed viewport of the client, and the user browses the image from left to right, the image located on the right side of the current area has a higher priority for caching, and the image closer to the current area also possesses higher priority. The cache area is a square, whose length is chosen by the longest side of the current monitor screen, as the smallest unit for caching. In addition to that, due to bandwidth limitations, the cache area cannot be too large. The multithreading approach is taken to improve cache efficiency.

### 3.4. Segmentation Data Model Design

The segmentation data from the EM image is obtained by human annotation or the automatic segmentation algorithms. Whether the segmentation data represents a neuron, an organelle (such as mitochondria), or other cellular structures (such as synapse), the essence is a region (set of points or a point) with some attributes. Besides, because of the considerable number of the segments, the way to store and retrieve segmentation data also needs to be resolved.

In this paper, the contour is used to represent each segmentation region to save storage space. Different cell structures are distinguished by the type attached to the contour. Additionally, it is taken as a unified data type called primitives, and the corresponding attributes (such as object id, object type, region centroid/object node, color, connection relationship between nodes within the neuron, and synaptic connection between neurons) are added to it. Sometimes, it may be necessary to represent two or more objects at the same point. For example, two cell structures have overlapping regions (such as a mitochondrion in a neuron), which is the first advantage of using contours to represent segmentation data instead of using voxels, which are represented by all pixels of the object. The second advantage is that it is helpful for 2D display and 3D rendering. The contour can be directly used for the display of plane figures and 3D rendering. If it is represented by voxels, the contour must be extracted, or a model must be built through voxels firstly before displaying or rendering. The third advantage is that the contour-based storage method does not have to store multiscale data. Unlike EM image data, this saves storage space further.

A preprocessing tool is provided for extracting primitives in large-scale segmentation data and obtaining the connection relationships between primitives. See “Appendix A” for specific details.

#### Block Storage

**Figure 5:**
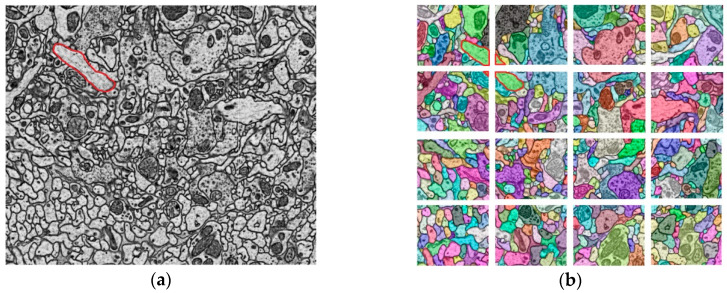
The designed segmentation data model. (**a**) The red line is the contour of the neuron, extracted from segmented data. (**b**) The contour in (**a**) is across four image blocks, so the whole contour data should be stored in each block.

When browsing primitives, there is a similar problem with browsing images. Only the primitives in the current viewport of the client should be displayed. The number of primitives is large, and the shape is irregular, which increases the difficulty of spatial retrieval. It is an extremely time-consuming process to traverse all primitives to get the current required primitives, and reading all primitives will also be limited by the memory size. As learning from the method of image storage, the primitives are stored in blocks. All primitives on the corresponding image block are stored as a separate primitive information file. If a primitive crosses multiple image blocks (such as the primitive indicated by the red outline in Figure 5a is distributed on the four image blocks in Figure 5b), it will be stored entirely in each block.

### 3.5. Segmentation Data Retrieval

**Figure 6:**
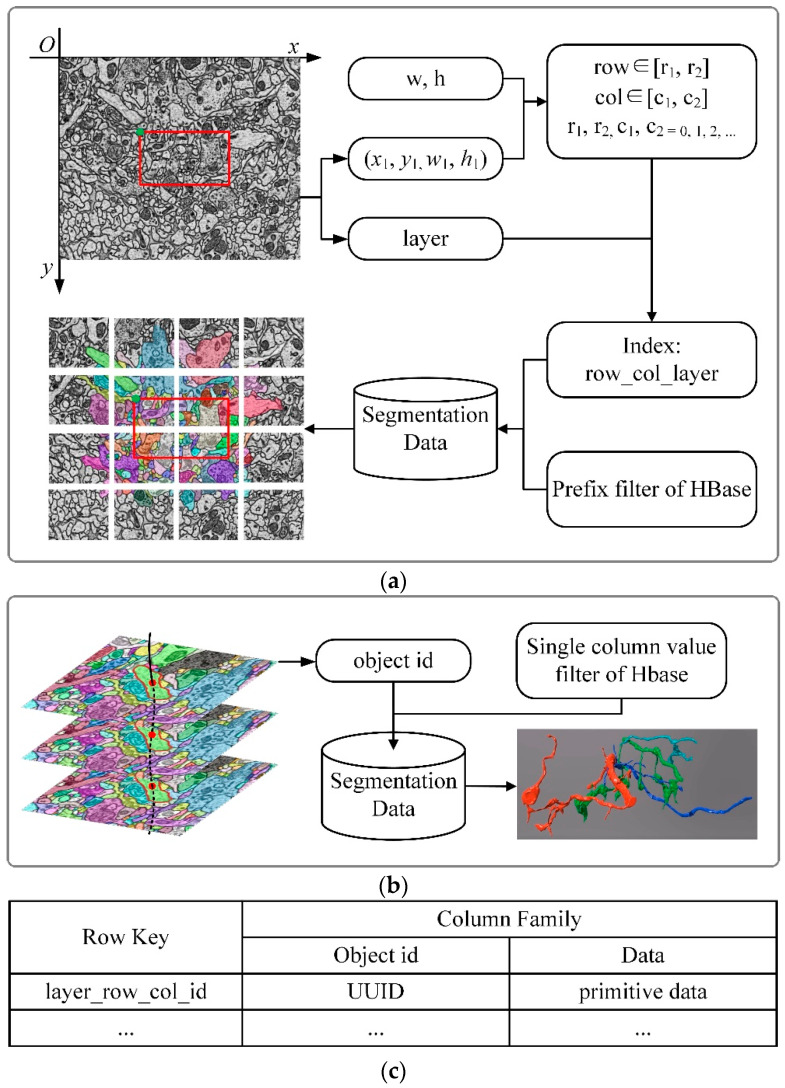
The flow of segmentation retrieval for 2D retrieval and 3D retrieval. (**a**) The flow of 2D retrieval. The meaning of parameters (w, h, x1, y1, w1, h1, layer) is the same as what is mentioned in Figure 3. As shown above, the advantage of block storage is that fewer primitives should be read, especially for large-scale images, where tens of thousands of primitives may exist. Now, the primitives in the current view need to be read. (**b**) The flow of 3D retrieval. A whole neuron consists of multiple contours in the sequence layer. A neuron has a unique id (object id in the figure), which is the parent id of every contour. The whole neuron can be obtained with the neuron id and the single-column value filter of HBase. (**c**) HBase table structure for storing primitives. The id in row key (layer_row_col_id) is the primitive’s id, which is unique in the table. The object id is the parent id of the primitives that compose it.

Due to the large number of primitives, compared with image data, the retrieval method of segmentation data is more complicated. When browsing segmentation data, two retrieval methods are needed, which are 2D retrieval for browsing the 2D view and 3D retrieval for getting a whole-cell structure, as shown in Figure 6. The HBase is used to store primitives, and the row key of primitives in HBase is named as “layer_row_col_id”, according to the method of block storage previously described. The prefix of the index “layer_row_col” is the index of the image block. UUID is employed as the id of the index to ensure that the id of primitives is globally unique.

#### 3.5.1. 2D Retrieval

As shown in Figure 6a,c, when browsing primitives on the 2D view, the indexes (layer_row_col) of the image blocks intersecting with the client viewport are generated as the same method in section “Image data retrieval”. Because primitives do not need to be downsampled, there is no “level” item in the index, and then use the prefix filter of HBase to get all the primitives with the indexes as “layer_row_col_*” (symbol “*” stands for the primitive id). After that, the primitives stored in the corresponding blocks are obtained, with the duplicates being removed.

#### 3.5.2. 3D Retrieval

Three-dimensional (3D) objects are composed of several 2D primitives. When a whole-cell structure (such as a whole neuron) needs to be displayed, all the 2D primitives that make up the object needs to be acquired, as shown in Figure 6b. It is an inefficient way to traverse all the id in row key (in Figure 6c) to get the needed primitives. In order to improve the retrieval efficiency, the single-column value filter of HBase is adopted to get the primitives by using the object id. All the acquired 2D primitives are stacked in sequence to form a 3D object.

## 4. Results

Based on the strategy proposed in Section 3, we have developed a data management module and introduced its main architecture and features in this section. We also make a test on our data management module, and it shows excellent real-time performance when handling large-scale data.

### 4.1. Outline of the Data Management Module

**Figure 7:**
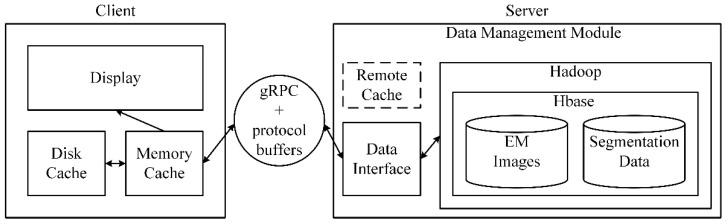
The architecture of the data management module. The data management module consists of two parts: an image cache module and a data storage and retrieval module. The image cache module has three levels: the memory cache, the disk cache, and the remote cache (the data at the server is considered as the remote cache). Data is stored in a distributed file system Hadoop, with a database HBase. The communication tools between clients and servers are gRPC and protocol buffers.

The architecture of the data management module is shown in Figure 7. The data management module mainly includes a data storage and retrieval module at the server and an image cache module at the client. On the server-side, the underlying file system is HDFS (Hadoop distributed file system), and the database is HBase for storing EM images, segmentation data, and other intermediate results. Furthermore, several methods are proposed based on the data characteristics to retrieve the required data rapidly and accurately. A data interface is designed to exchange data between clients and servers. The image cache at the client is a three-level cache module, and the data at the server is considered as the third level (remote cache). By establishing first-level and second-level caches in the memory, and the local disk of the client, the time delay for the client to obtain image data is reduced. The client and the server communicate with each other through the network communications engine gRPC (a high-performance, open-source universal RPC framework) and protocol buffers (Google’s language-neutral, platform-neutral, extensible mechanism for serializing structured data).

The data management module is used to manage large-scale neural structure data in the field of connectomics, mainly for EM images and its segmentation results. The data storage and retrieval module on the server-side of the management module are written in Java 8 and can run both on Microsoft Windows (64 bit) and Linux, because of the cross-platform feature of Java. For convenience use, the storage and retrieval module is packaged into a jar package. The image cache module on the client-side is written in C++ 11, it has been thoroughly tested on Microsoft Windows (64 bit) and can also run on Linux with a simple modification.

### 4.2. Large-Scale Data Support, Scalability, and Data Security

At present, managing large-scale neural structure data is one of the bottlenecks in reconstructing the connectome of the brain. The purpose that we develop this tool is to manage large-scale data effectively so that researchers can mine valuable information. The electron microscope image data of the full adult fly brain released by Google and Janelia Research Campus et al., after compression processing, is still 26 TB [22], which is only the EM images. Segmentation results and other intermediate data will also be generated during the reconstruction process. With the advancement of technology and the needs of scientific research, the volume of data will grow more significantly in the future (PBs or even EBs). To this end, the fully developed and widely used distributed file system Hadoop and distributed database HBase are employed to store and retrieve massive data. Another advantage of the distributed architecture built with Hadoop and HBase is that it supports the excellent scalability. At the same time, redundant copies of the data are kept in Hadoop clusters to prevent data from being lost or corrupted during storage or processing. The security and integrity of the data are guaranteed to a great extent.

### 4.3. Real-Time Data Access

Meeting the storage needs of large-scale data is only the first step in data management. How to retrieve the required data from a large-scale database and achieve the purpose of real-time access is also one of the problems that need to be solved. HBase integrates many convenient and fast retrieval interfaces, which can retrieve data in multiple ways. It uses key-value pairs for retrieving, storing, and accessing data in a columnar approach. Based on the nature of the data, combined with multiple retrieval interfaces of HBase and the sort mode, the purpose of fast retrieval is achieved by designing a reasonable key and a data access strategy for various data.

On the premise of achieving fast retrieval, reading the data, and transmitting them from the server to the client also take some time. To reduce the delay time of data acquisition at the client, and finally achieve the purpose of real-time access, in this paper, a three-level cache module on the client-side was developed to cache EM images. When browsing the images’ area of interest, the image data (different scales and sequence images) near the area will be simultaneously cached. Additionally, the cache priority of the image data is set: both the data closer to the browsing area and that in the browsing direction has higher priority. Through such a caching strategy, the required data is cached for subsequent browsing, which makes the data acquisition more purposeful. At the same time, in order to reduce the time consumption of data transmission and save storage space, the JPEG compression technology is used to store and transmit data. The data is decompressed at the client to meet the needs for real-time data access further. Video S1 shows browsing EM images in real time and is available in “Supplementary Materials”, and please refer to “Appendix A” for specific information about the data in the video.

### 4.4. Data Sharing

A standard interface is designed for the client to access data from the server, combined with gRPC and protocol buffers. Based on the interface, developers can design client applications and use the server-side of the module as a backend to manage massive data. By adding other public databases, the server-side of the module can manage the public databases in the field of connectomics, mainly for EM images. Users acquire shared datasets through the standard interface. Users can also make datasets by using our tools and store them in a public database or the distributed storage module designed by us to share their data. For specific information on interface definition and implementation, please refer to “Appendix A”.

### 4.5. Making Datasets and Testing

Before using our tools, Hadoop and HBase need to be deployed on distributed cluster servers. A tool is provided to generate the dataset, and it is integrated with the data storage and retrieval module. For the format of the dataset, please refer to the section “image data model design” in the methods. A testing dataset was made to evaluate the performance of our tool. The tool that generates datasets are integrated into the data management module with a graphical user interface (GUI); for specific details and tutorial, please refer to “Appendix A”. The sequential image data used to make the dataset has 50 layers and is the 8-bit grayscale image. The size of each layer is 470,000 × 425,000 pixels, and it is stitched by 50 × 50 images whose size is 9400 × 8500 pixels. The resolution of the image is 5 nm × 5 nm × 50 nm, and the size of the whole test data is 2.35 mm × 2.125 mm × 2.5 um. The uncompressed serial image is featured with a data volume of 9.08 TB. Seven servers are configured with Linux systems into a distributed cluster. To prevent data loss or damage, the number of data redundancy in Hadoop is three. It took about 165.9 h (6.9 days) to generate the dataset on one computer, and multiple computers can be used to speed up the process. Finally, the total volume of the test dataset is 24.27 TB (contains multiresolution EM images and three copies in Hadoop), with the JPEG compression format. The performance of our tool is tested in two ways. One is to measure the time for acquiring the image under different browsing modes and calculate the average access time, while the other is to test the concurrent access performance of the tool.

**Figure 8:**
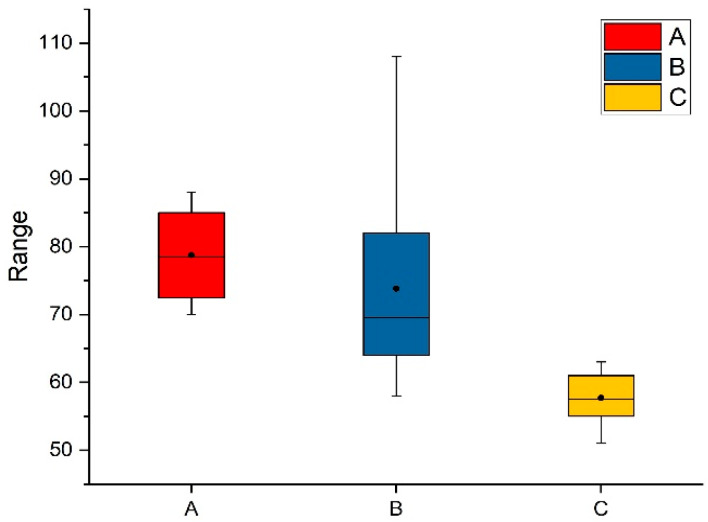
The average access time of image data. The unit of the *Y*-axis is in milliseconds. (**A**–**C**) represent the average access time for the client to obtain image data under different browsing situations. During testing, extreme cases of brute force browsing were ruled out.

The first is to test the average access time of image data (image block size is 2048 × 2048 pixels) in different browsing modes. The data access time refers to the time elapsed since the server receives the index of the image block and performs a retrieval operation until the image block data is read into the memory of the server. The test results are shown in Figure 8, and the unit of the *Y*-axis is in milliseconds. Figure 8a shows the average access time calculated by browsing data between different scaling factors of the specified layer. Figure 8b shows the average access time calculated by browsing different areas of the layer under the specified scaling factor. Figure 8c shows the average access time calculated by browsing the different layers of the sequence image at a specified scaling factor. From Figure 8, it can be found that the most data access time is in 90 ms, and then it can be concluded that when browsing between different scaling factors at a particular layer, it takes more time to acquire images; browsing different layers at a particular scaling factor takes less time to acquire images.

The second is to test the concurrent access performance of the data management module when 50 people browse the same dataset at the same time. Under the gigabit bandwidth, the network transmission rate for each user at the client-side is about 2 MB/s, while the rate at the server-side reaches about 100 MB/s, which has reached the fastest transmission rate under the gigabit bandwidth. With the compressed data, there is no delay when users browse images, which completely meets the needs of users under normal browsing conditions.

Besides, our data management method is also used for the study of the mitochondrion to decode brain activities [38] in recent research.

## 5. Discussion

Mining valuable information from data in the field of connectomics will help us explore our brain. However, as the volume of tissue blocks to be reconstructed increases, the resolution of the electron microscope increases, and the thickness of sequence slices decreases, the volume of data grows more massive prevalently. Facing such large-scale data, providing useful management tools for researchers is the basis of this work. The purpose of developing this management module is to solve the problem of managing large-scale neural structure data, so as to help researchers to mine valuable information. Based on the standard application program interface (API), the server-side of the module can also be taken as the back end of other similar software or a public database to manage shared datasets.

The storage models and retrieval methods are mainly designed for the EM images and segmentation results. With the distributed frameworks (Hadoop and HBase), while solving the storage and retrieval problems of such big data, the scalability of this module is also improved. As data increases, the storage pressure can be eased by adding server nodes, which is simple and easy to do. In addition to that, the redundant copies are conducive to ensuring the security of the data. At the same time, combined with the filters of HBase, a standard data interface and retrieval methods are designed to achieve the purpose of fast retrieval. The retrieval method has constant time complexity O(1). With the increase of data volume, the retrieval speed will not be affected. At the client-side, to reduce the delay time of data acquisition further, a three-level cache module is designed for EM images, which improves the performance of our software tools. With the fast retrieval method and the image cache module, our tool is featured with excellent real-time performance when accessing data. The design of the storage structure, the retrieval method, and the cache module are to cope with the increasing volume of data, for making our tool universal. Besides, it will not be affected by the volume of data. The universality of our tool is also shown in the application of other data, such as optical microscope data and MRI data.

Of course, the tool will be refined. In the future, in order to further improve the user experience, reduce the pressure on server requests, and promote data sharing between users, the p2p network structure [39,40,41] is intended to apply to our tools. Moreover, expecting that through the continuous improvement of software tools, the reuse of data in the field of connectomics and sharing between users can be promoted so that the potential value of the data can be maximumly excavated. We also hope to make a contribution to the development of connectomics with the majority of researchers.

## 6. Conclusions

The reconstruction of the connectome will help researchers to explore the brain. With the scale of data grows, which generated during the reconstruction process, managing the large volume data is a challenge. A massive data management tool is developed in this paper, based on a distributed architecture. Firstly, on the server-side, combining with Hadoop and HBase, many data management strategies are put forward to solve the problem of large-scale data storage and retrieval. Secondly, in order to further reduce the time delay of data acquisition, an image three-level cache module is proposed on the client-side. Then, a standard interface is designed for communicating between the server and the client. Finally, by making tests on the data management tools, it shows excellent real-time performance when handling large-scale data. Additionally, the server-side can be used as a backend of other similar software or a public database to manage shared datasets, showing strong scalability. However, with the increasing demand of neuron circuit reconstruction, the pressure of data management grows in many ways, such as Increasing types of data to be managed, further improving the experience of users, making the concurrent processing ability of the server better and so on, so that will be the main direction of our future work.

## Figures and Tables

**Table 1:** Reconstruction results over the years

Year	Sample	Volume (um^3^)	Reconstruction State
2011	Visual cortex of mice [15]	120 × 80 × 130	Sparse ^1^
2013	Mouse inner reticulum [16]	132 × 114 × 80	Sparse
2015	Rat cortex/retina [17]	44 × 60 × 141/93 × 60 × 93	Sparse
2016	Rat visual thalamus [18]	400 × 600 × 280	Sparse
2017	Songbird X Area [19]	98 × 96 × 115	Sparse
2018	Drosophila brain [20]	750 × 369 × 286	Sparse
2018	Songbird X Area [21]	98 × 96 × 115	Dense ^2^
2019	Drosophila brain [22]	995 × 537 × 283	Dense

^1^ The sparse state means to reconstruct a few neurons of interest. ^2^ The dense state means to reconstruct most of the neurons in a tissue block.

**Table 2:** The transformation between magnitude and level.

Magnitude	Level
mag ^1^ > 50%	0
50% ≥ mag > 25%	1
25% ≥ mag > 12.5%	2
12.5% ≥ mag > 6.25%	3
2^-n+1^ ≥ mag > 2^-n^	n-1
2^-n^ ≥ mag	n (if n is the maximum level.)

^1^ Abbreviation of magnitude.

## Supplementary Materials

The following are available online at https://www.micro-visions.org/git/MiRA-Open/DataManager/src/branch/master/videos. Video S1: Showing browsing EM images in real time.

## Appendix A

The full source code of the data management module and a demo shown in MiRA (a software developed by ourselves) are available at https://www.micro-visions.org/git/MiRA-Open/DataManager.git, with a tutorial of how to use the module and generate datasets in README.md.

The definition and implementation of the standard interfaces is available at https://www.micro-visions.org/git/MiRA-Open/DataManager/src/branch/master/DataManager/src/main/proto and https://www.micro-visions.org/git/MiRA-Open/DataManager/src/branch/master/DataManager/src/main/java/org/casia/mira/server.

For adding another data source in the field of connectomics, first of all, the public database should provide the application program interface (API) for getting data, better written in Java. Then, according to the usage of the API, modify the function “getImages” in https://www.micro-visions.org/git/MiRA-Open/DataManager/src/branch/master/DataManager/src/main/java/org/casia/mira/server/ImageServerIO.java to add a new data source.

The data shown in Video S1 is available at https://neurodata.io/data/kasthuri15/.
